# Subchronic Toxicity Study of Alternanthera philoxeroides in Swiss Albino Mice Having Antioxidant and Anticoagulant Activities

**DOI:** 10.1155/2022/8152820

**Published:** 2022-07-13

**Authors:** Shahad Saif Khandker, Morshed Alam, Forhad Uddin, Ummay Mahfuza Shapla, Nafisa Lubna, Tanusree Amy Mazumder, Mahfuza Marzan, Milon Mondal, Md Ibrahim Khalil, Nurul Karim, Md Salman Shakil, Md Sakib Hossen

**Affiliations:** ^1^Department of Biochemistry and Molecular Biology, Jahangirnagar University, Dhaka 1342, Bangladesh; ^2^Gonoshasthaya Samaj Vittik Medical College, Savar, Dhaka 1344, Bangladesh; ^3^Department of Biochemistry and Molecular Biology, Bangabandhu Sheikh Mujibur Rahman Science and Technology University, Gopalganj, Dhaka 8100, Bangladesh; ^4^Department of Medicine and Surgery, Zainul Haque Sikder Women's Medical College and Hospital, Monica Estate, Western Dhanmondi, Dhaka 1209, Bangladesh; ^5^Department of Microbiology, Jahangirnagar University, Dhaka 1342, Bangladesh; ^6^Department of Pharmacy, Bangabandhu Sheikh Mujibur Rahman Science and Technology University, Gopalganj 8100, Bangladesh; ^7^Department of Mathematics and Natural Sciences, Brac University, 66 Mohakhali, Dhaka 1212, Bangladesh; ^8^Primeasia University, Department of Biochemistry and Molecular Biology, Banani 1213, Dhaka, Bangladesh

## Abstract

*Alternanthera philoxeroides*, a tropical herb and edible vegetable, has been popular as a medicinal plant. Applying *in vitro* approach, we initially attempted to assess the phytochemicals, bioactive chemicals, as well as antioxidant and anticoagulant activities of this plant. Following that, the *in vivo* toxicological effects of methanolic extracts of *A. philoxeroides* using different doses on the kidney, heart, lung, liver, stomach, brain, and blood of female Swiss Albino mice were investigated. We estimated phytochemicals content as well as antioxidant activity through DPPH, NO, CUPRAC, and reducing power assays, followed by the anticoagulant activities of PT  and aPTT  and bioactive compounds using HPLC. To confirm the biocompatibility of *A. philoxeroides* extracts, histopathological and hematological parameters were examined in a mice model. Total phenol, flavonoid, and tannin content in *A. philoxeroides* was 181.75 ± 2.47 mg/g, 101.5 ± 3 .53 mg/g, and 68.58 ± 0.80 mg/g, respectively. Furthermore, the HPLC study confirmed the presence of four phenolic compounds: catechin, tannic acid, gallic acid, and vanillic acid. The methanolic extract of *A. philoxeroides* showed considerable antioxidant activity in all four antioxidant assay methods when compared to the standard. In comparison to ascorbic acid, *A. philoxeroides* also demonstrated a minor concentration-dependent ferric and cupric reduction activity. *In vivo* evaluation indicated that *A. philoxeroides* extracts (doses: 250, 500, and 1000 mg/kg) had no negative effects on the relative organ or body weight, or hematological indicators. Our study concluded that *A. philoxeroides* had significant antioxidant and anticoagulant activities and demonstrated no negative effects on the body or relative organ weight, histopathological, and hematological indices in the mouse model.

## 1. Introduction

Free radicals are one of the most important factors in the pathophysiology of different diseases, i.e., arthritis, atherosclerosis, diabetes, Alzheimer's disease, cancer, and other oxidative stress [1]. Besides playing a role in causing various diseases, free radicals are primarily responsible for the aging process and injury to cells [2–4]. Abnormal blood coagulation activity or thrombosis is another crucial health complication that is related to different diseases including atherosclerosis, liver disease, and diabetes [5–7]. Conventional anticoagulant drugs (such as heparin and warfarin) are used to treat thrombosis; however, these drugs have many side effects, including allergic reactions, alopecia, and major bleeding skin reactions [8, 9]. To overcome the limitations of conventional anticoagulant drugs, safe and effective anticoagulation should be developed.

Herbal medicines are gaining special attention in this aspect, and their potential roles in Bangladesh's diverse locations are being researched [10]. Polyphenolic chemicals, which make up a substantial portion of the active ingredients in these plants' extracts, have been found to have numerous protective properties, including antioxidant, anti-inflammatory, anticoagulant, and antiproliferative activity [11–16].

**Figure 1:**
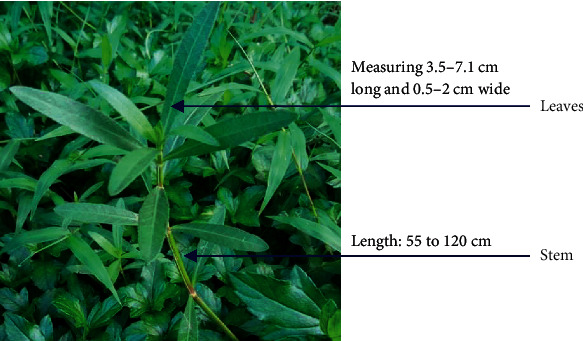
Distinguishing features of *Alternanthera philoxeroides*.

*Alternanthera philoxeroides* (alligator weed) is a “perennial stoloniferous herb” that grows in aquatic to terrestrial environments (Figure 1) [17]. In Bangladesh, the vernacular name of this plant is “Malancha shak” and is sold as a vegetable in the local market [16]. It has been very popular in different health complications including female diseases, night blindness, coughing up blood, hematuria, measles, cold and pyrexia, stranguria with turbid urine, encephalitis B, anthracia, eczema, venomous snake bite, and furunculosis, from the past until now among village people and folk doctors [17, 18]. Pamilla et al. used the GC-MS method to identify the phytochemical components, which may give insight into its usefulness in traditional medicine [19]. Fang et al. also discovered two novel anticancer chemicals, namely, alternanthin B and N-trans-feruloyl-3,5-dimethoxytyramine from the ethanolic extract of this plant [20]. Despite the wide range of uses for this plant, there appears to be a scarcity of knowledge concerning its toxicity.

The therapeutic and antioxidant capabilities of *A. philoxeroides* have previously been shown [10, 21–25]. However, not much information and research on the antioxidant contents and activities of *A. philoxeroides* grown in Bangladesh can be found. To establish *A. philoxeroides* as a useful therapeutic alternative, we investigated its antioxidant and anticoagulant properties, as well as the identification of several important phytonutrients. After that, we devised our study procedure to investigate the potential subchronic toxicity of the methanolic plant extract using a histopathological and hematological investigation in the Swiss Albino mouse model. We selected methanol as an extraction solvent since it can dissolve both polar and lipophilic bioactive molecules. Furthermore, because this solvent is easily evaporated, we acquire the most bioactive components from the extract by limiting the loss of substances owing to high temperature [26].

## 2. Methods and Materials

### 2.1. Extraction and Processing of Herbs

The entire plant of *A. philoxeroides*, including leaves, stem, and roots, was taken from Savar (23.8583°N 90.2667°E), Dhaka, Bangladesh, and washed finely to eliminate any earthy debris, decaying leaves, and contaminants. The collected plants of *A. philoxeroides* (Plant ID: JUH 10070, accession number from Botany Department, Jahangirnagar University, Bangladesh) were then sun-dried and powdered with an industrial grinder followed by the preparation of methanolic extract by adding 500 mL of 99.9% methanol to 100 g of the powder. The mixtures were then kept in a shaker (IKA400i, Germany) at 30° C and 150 rpm for 72 h followed by filtration. The filtrated extract was then dried out at 40°C to evaporate the methanol. The extracted material had an approximate crude yield of 8% w/w and was kept at −20°C until further use.

### 2.2. Reagents and Chemicals

All the reagents and substances utilized in this experiment were extremely unadulterated and pure. Folin–Ciocalteu reagent (Merck Co., Darmstadt, Germany), gallic acid, tannic acid, vanillic acid, catechin, rutin, quercetin, TPTZ, 2, 2-diphenyl-1-picrylhydrazyl (DPPH), neocuproine, sodium nitroprusside, Griess reagent (Sigma-Aldrich, St. Louis, MO, USA), hemostat thromboplastin SI reagent, and hemostat aPTT-EL reagent (Human Diagnostic, Germany) were mainly used for the assays that were required.

### 2.3. Phytochemical Analysis

Total polyphenol content was calculated using an established approach that relies on Folin–Ciocalteu mixture [27]. Chang and Yang's aluminum chloride colorimetric test methodology was used to assess total flavonoid concentration [28] with slide modification, and the tannin level was calculated using the Folin–Ciocalteu approach [22]. A brief explanation of these techniques has been provided in Supplementary File 1.

### 2.4. HPLC Profiling of Phenolic Substances

To detect the phenolic chemicals in *A. philoxeroides* extract, we used a slightly modified version of the existing technique [29, 30]. In brief, an extract solution of *A. philoxeroides* was produced in methanol and filtered using a 0.45 m syringe filter (Sartorius AG, Germany). The filtrate was put into an HPLC system (specification: “SPD-20AV, serial number: L20144701414AE, Shimadzu Corporation, Kyoto, Japan)” with a UV detector (specifications: “SPD-20AV, serial number: L20144701414AE, Shimadzu Corporation, Kyoto, Japan).” HPLC column specifications were “Luna Phenomenex C18 100A (150 × 4.60 mm, 5 *μ*m).” A linear gradient at a flow rate of 0.5 mL/min was maintained throughout the 35-minute duration of the analysis [29].

The mobile phase was composed of solvent A (HPLC grade methanol with 0.1 percent phosphoric acid) and solvent B (HPLC grade water with 0.1 percent phosphoric acid). The following technique was performed to obtain the elution of the column: from 0 to 10 minutes, the concentration of solvent B grew from 35% to 55%; from 10 to 25 minutes, the concentration of solvent B increased to 62 percent; from 25 to 30 minutes, the concentration of solvent B increased to 85 percent; and the final composition remained constant until 35 minutes [31]. The solvents utilized were of HPLC grade. The detecting wavelength was set between 200 and 450 nm, and particular inspection was conducted at 265 nm. The timescales of analytes were matched to reference standards to distinguish phenolic and flavonoid groups. All standards utilized to determine phenolic and flavonoid chemicals were pure and unadulterated.

### 2.5. Antioxidant Activity

Antioxidant ability of *A. philoxeroides* was estimated through the 2,2-diphenyl-1-picrylhydrazyl (DPPH) radical scavenging activity [32], nitric oxide (NO) scavenging capacity [33], cupric reducing antioxidant capacity (CUPRAC) [34], and ferric reducing antioxidant power assay (FRAP) [35] with slight modification. In Supplementary File 1, a brief description of these approaches is presented.

### 2.6. Anticoagulant Activity Analysis

The anticoagulant activity of *A. philoxeroides* extract and the biologically active compounds identified through the HPLC method were evaluated individually and compared.

#### 2.6.1. Collection of Plasma Samples

The blood of a healthy adult female was collected in PT tubes containing sodium citrate (3.2%) to avoid the natural process of blood coagulation. Through the centrifugation process (3000 rpm, 10 min), the plasma supernatant was separated from the whole blood and was stored in another container.

#### 2.6.2. Determination of Prothrombin Time (PT)

Prothrombin time was determined following the previous method [36] with slight modification. Various concentrations (250–1000 *μ*g/mL) of *A. philoxeroides* extract solution and a single concentration (10 *μ*g/mL) of each bioactive compound detected in HPLC analysis were prepared for this test as a sample. The reagent (consisting of rabbit brain extract and sodium azide) and buffer solution (consisting of CaCl_2_ and sodium azide) were mixed at equal volume and prewarmed at 37°C primarily. Plasma and sample solutions were mixed in equal volume (50 *μ*L) and prewarmed at 37°C for 2 minutes. Finally, to the mixture of plasma and sample, 200 *μ*L of reagent mixture was poured, and the clotting time was documented. The clotting time of blood plasma diluted with deionized water in the place sample was used as the control.

### 2.7. Activated Partial Thromboplastin (aPTT) Time Computation

aPTT was measured following the previous method [36] with some slight modifications. Different concentrations (250–1000 *μ*g/mL) of *A. philoxeroides* extract solution and a single concentration (10 *μ*g/mL) of each biologically active compound detected in HPLC analysis were prepared separately as a sample and in prewarmed test tube plasma (50 *μ*L) and sample (50 *μ*L) solution was blended properly. 100 *μ*L of reagent I was poured into the formulation and was incubated for 3 min at 37°C. Prewarmed (at 37°C for 2 min) reagent 2 (consisting of CaCl_2_, sodium azide, salts, and stabilizers) was finally added to the chemical mixture, and the clotting duration was documented. The clotting time of blood plasma mixed with deionized water in the place sample was used as the control.

### 2.8. Experimental Animal

Female Swiss Albino mice (20–25 g) were procured from the Department of Pharmacy, Jahangirnagar University, Bangladesh-1342, and the experiment was conducted in the Department of Biochemistry and Molecular Biology at Jahangirnagar University. The animals were kept in sterile plastic crates with soft woodchip matting and were trained on a 12-hour day-night cycle. The animals were cared for under standard laboratory settings, which included a temperature of 22 ± 2°C, relative humidity of 40–56%, and a 12-hour light/dark artificial photoperiod. They were fed well and given access to clean water on a regular basis. All animals were allowed a 7-day acclimation period in the lab prior to the study. The experimental setup was approved by Jahangirnagar University's Biosafety, Biosecurity, and Ethical Committee [Ref No: BBEC, JU/M 2021 (4)2].

### 2.9. Experimental Groups

To conduct our in vivo studies, we randomly allocated the mice into four groups of six mice each. Animals in Group I (Control) were fed a routine diet (no *A. philoxeroides* dosage) for 28 days, while those in Groups II, III, and IV were treated with *A. philoxeroides* extract (250 mg/kg, 500 mg/kg, and 1000 mg/kg, respectively) dissolved in saline water via oral gavage. All animals were served a regular laboratory meal and were allowed to drink water whenever they wanted.

The body masses of the mice were monitored weekly during the trial. To identify any indicators of disorders, the animals were also inspected during their eating and drinking activities for behavioral issues. The animals were starved for 16 hours before being sacrificed. Blood was drawn from the inferior vena cava and kept in EDTA-containing tubes, and the chosen organs were collected and stored in formalin (10%)-containing tubes for hematological and histological investigations shortly after the execution.

### 2.10. Influence on Body Weight and Relative Organ Weight

The animals' body weight growth was tracked every seven days throughout the experiment. The heart, liver, kidneys, lungs, stomach, and brain were all collected and weighed immediately after euthanasia. The relative body weight was computed by dividing the individual body weight of each mouse by the individual body weight of each mouse.

### 2.11. Histopathological Analyses

The liver, kidney, lung, stomach, brain, spleen, and heart were all taken out and cleaned in saline solution for histological inspection. The organs were then buried in a neutral formaldehyde solution at a concentration of 10%. After that, the paraffin was applied to the preserved organ tissues. Using a previously known technique, the paraffinized tissue specimens were sliced into 5 *μ*m thick slices and stained with hematoxylin and eosin (H&E) for histological examination. A standard spectrum fluorescent microscope (specification: “Olympus DP 72”) with a digital camera attached (specification: “Olympus, Tokyo, Japan”) was used to capture histology images.

### 2.12. Hematological Analyses

Hematological parameters such as hemoglobin (Hb), WBC count (TC), RBC count, total platelet count (PC), different WBC Count as in neutrophils, lymphocytes, monocytes, eosinophils, basophil, hematocrit (HTC/PCV), mean corpuscular volume (MCV), mean corpuscular hemoglobin concentration (MCHC), mean corpuscular hemoglobin (MCH),, red cell distribution width (RDW), mean platelet volume (MPV), platelet distribution width (PDW), procalcitonin (PCT), and total cir. eosinophil count (TEC) were analyzed in all animal groups using an automated hematology analyzer (model: “8000i, Sysmex, Japan”).

### 2.13. Data Interpretation

All of the assessments were repeated three times, and the data were compiled in Microsoft Excel 2016 (version 2108). The data were presented as the mean ± SD (standard deviation). GraphPad Prism 8 (version 8.4.3 (686)) was used to evaluate quantitative data and create a graphical presentation. The data sets were analyzed using one-way ANOVA and Dunnett's multiple comparison. The significance level was set at P 0.05.

## 3. Result

### 3.1. Phytochemicals

Table 1 summarizes the concentrations of bioactive phenols, flavonoids, and tannins in *A. philoxeroides.*

### 3.2. HPLC Analysis

A total of six phenolic standards tannic acid, catechin, gallic acid, vanillic acid, quercetin, and rutin were used in our study. Among them, tannic acid, catechin, gallic acid, and vanillic acid were identified within the sample by HPLC analysis. The detection was conducted based on the retention time (Table 2).

### 3.3. Antioxidant Activities

**Figure 2:**
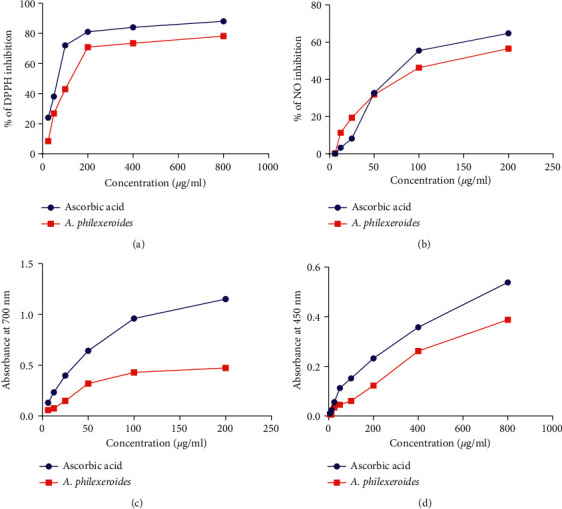
This figure shows (a) DPPH free radical scavenging activity, (b) NO free radical scavenging activity, (c) reducing power capacity, and (d) cupric reducing antioxidant capacity of the methanolic extract of *A. philoxeroides*.

In all four antioxidant assay methods (i.e., DPPH free radical scavenging activity, reducing power activity, NO free radical scavenging activity, and cupric reducing antioxidant capacity), we found a significant antioxidant activity of *A. philoxeroides* compared to the ascorbic acid (Figure 1). The IC_50_ values of *A. philoxeroides* extract for DPPH and NO free radical scavenging activity were found as 116.63 *μ*g/ml and 176.74 *μ*g/ml, respectively (Figures 2(a) and 2(b)).

The ferric and cupric assays, on the other hand, were used to evaluate *A. philoxeroides'* antioxidant reducing power. In comparison to ascorbic acid, our data imply that *A. philoxeroides* has a decent concentration-dependent ferric and cupric reducing activity (Figures 2(c) and 2(d)).

### 3.4. Anticoagulant Activity

The clotting time of blood plasma was investigated to be increased for both the PT and aPTT tests in the presence of *A. philoxeroides* in comparison to the control clotting time. With the increasing concentration of *A. philoxeroides,* the clotting time was found to be increasing (Table 3).

### 3.5. Anticoagulant Activity of Bioactive Compounds

Apart from assessing the anticoagulant activity of the sample extract (*A. philoxeroides*), the anticoagulant activity of the bioactive components identified through HPLC analysis was also assessed (Table 4).

### 3.6. Physical Appearance, Body Weight, and Relative Organ Weight Profiling

Neither the normal nor the positive control animals showed any indicators of toxicity, such as slight tremors, depression, soft feces (mild diarrhea), or dyspnea, during the study periods.

**Figure 3:**
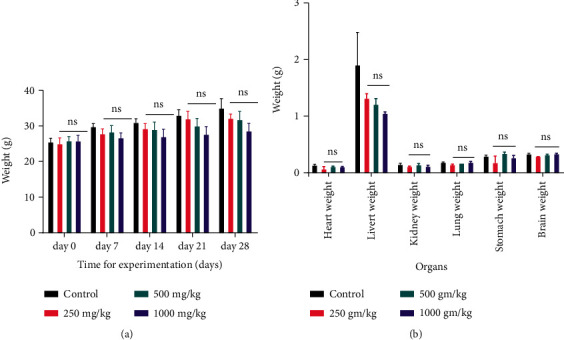
Effect of the methanol extract of A. philoxeroides on (a) body weight and (b) relative organ weight. Data are presented as the mean ± standard deviation (*n* = 6). No significant difference was calculated for tests in the A. philoxeroides methanol extract (250 mg/kg, 500 mg/kg, and 1000 mg/kg) treated groups compared to control were compared with the control group (*p* < 0.05) using one-way ANOVA followed by Dunnett's multiple comparison. Here, ns= nonsignificant.

When the three dosages of *A. philoxeroides* methanol extract (250 mg/kg, 500 mg/kg, and 1000 mg/kg) were compared to the control, there was no significant difference in body weight growth. The body weight steadily grew throughout the course of the trial, although there were no significant differences between the first and third weeks (Figure 3(a)).  Figure 3(b) is a visualization of no discernible change in the relative organ weight of the principal bodily organs.

### 3.7. Histopathology

**Figure 4:**
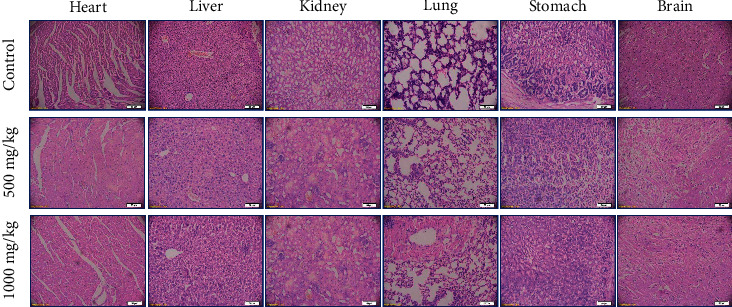
Histological images of heart, liver, kidney, lung, stomach, and brain tissue of A. philoxeroides (500 mg/kg and 1000 mg/kg) treated and controlled mice. Sections of the heart, liver, kidney, lung, stomach, and brain tissue were harvested from the female Swiss Albino mice 28 days of A. philoxeroides (500 mg/kg to1000 mg/kg) after treatment. Test samples were processed with hematoxylin and eosin (H and E) stain. Images were taken at 20X magnification. No pathological changes were observed in the case compared to the control.

After 28 days, histological examination of the heart, liver, kidney, lung, stomach, and brain revealed no architectural or degenerative changes as compared to the control. The photomicrographs for the two highest concentrations (500 mg/kg and 1000 mg/kg) are shown in Figure 4.

### 3.8. Effects on Hematological Parameters

The results of selected doses of *A. philoxeroides* extract on different parameters regarding hematology are tabularized in Table 5. In comparison with the control group, the outcomes from hematological analysis only exhibited a statistically substantial reduction in neutrophil count, while all other parameters remained unaffected by the extract.

## 4. Discussion

In almost all plants, secondary metabolites or phytochemicals such as polyphenols, flavonoids, and tannins are found although the quantity may be different. However, these compounds are very powerful antioxidants besides being a contributor of color, flavor, and functional features of a plant. They are able to reduce oxidative stress by arresting free radicals as they have the ability to donate electrons. Free radicals such as singlet oxygen molecules, hydroxyl radicals, superoxide radicals, hydrogen peroxide, and other prooxidants can be decreased by the activity of these compounds [37–39]. Phenolic compounds can also chelate metals and disrupt chain reactions besides reducing free radicals, and a correlation between total phenol and antioxidant activity was confirmed in the previous research [40, 41]. Polyphenols can work as a growth inhibitor of various pathogens including different kinds of bacteria, fungi, and even some viruses as well [42, 43]. In another case, dietary polyphenols are found to be effective in inflammatory bowel disease [44]. Polyphenol consumption also helps to minimize the risk of diabetes, cardiovascular diseases, cancer, neurodegenerative disorders, and obesity [45–47]. Flavonoids are another bioactive compound that can work as a modulator of immune functions [48]. It can also prevent cell proliferation, invasion or metastasis, angiogenesis, and inflammation. The molecular targets of these mechanisms can be repressed by flavonoids. Thus, it might be beneficial in the prevention of cancer [49, 50]. Tannin is also a bioactive component which is effective as an antibacterial, antifungal, and antiviral agent. It can even play a role in reducing the mutagenic and carcinogenic activity of various mutagens and carcinogens [51].

In this study, *A. philoxeroides* was found as a good source of polyphenols, flavonoids, and tannins (Table 1). Total phenol, flavonoid, and tannin content was 181.75 ± 2.47 mg/g, 101.5 ±  3.53 mg/g, and 68.58 ± 0.80 mg/g, respectively. Using the same approach, Ramproshad et al. found that the total phenol, flavonoid, and tannin contents of *A. philoxeroides* were 109.25 ± 0.43 mg/g, 78.52 ± 0.22 mg/g, and 15.16 ± 0.09 mg/g, respectively [52]. Polyphenols, flavonoids, and tannin content in A. philoxeroides, on the other hand, ranged from 0.0053 mg/g to 12.4 mg/g, 0.0187 mg/g to 3.2 mg/g, and 0.2045 mg/g to 5.6 mg/g, respectively, according to the previous studies [10, 53]. The considerable variations in these phytochemicals identified in *A. philoxeroides* extract may be due to the solvents used in the extracts since higher polarity solvents tend to yield higher quantities of polyphenolics [54]. In addition, Siatka et al. (2010) demonstrated that disparities in polyphenol content across the journal literature might be attributed to the difference in the harvesting period and geographical region in which the plant grows as flavonoid content is regulated by multifactorial environmental stimuli [55].

Using HPLC, we uncovered the following four phenolic components in *A. philoxeroides'* methanolic extract: tannic acid, gallic acid, catechin, and vanillic acid (Table 2). On the other hand, Kumar et al. (2015) identified chlorogenic acid, epicatechin [a, catechin with (2R,3 R)-configuration], caffeic acid, umbelliferone, rutin, quercetin, kaempferol in methanolic leaf extracts of *A. philoxeroides* [56]. Catechin was found in methanolic extracts of *A. philoxeroides* in these investigations as a common phenolic component which has antiplatelet and anticoagulant activity [12]. It can also be useful for treatment or to prevent Alzheimer's disease [57]. Tannic acid shows anticancer activity and helps to reduce cardiotoxicity [58]. It also plays a role as an anti-inflammatory, antioxidant, and antiproliferative agent [59]. Gallic acid is an antioxidant with anticancer properties, as well as having neuroprotective properties [13, 15]. While vanillic acid works as a hepatoprotective agent against liver injury [60]. It also shows anti-inflammatory activity and improves ulcerative colitis [61].

Free radical scavenging activity or antioxidant property of *A. philoxeroides* was determined by different tests such as DPPH radical scavenging activity, FRAP assay, reducing power activity, total antioxidant capacity, cupric reducing antioxidant capacity, and NO scavenging activity. DPPH is one of the most common and useful assay methods by which the radical scavenging activity of a sample can be measured. DPPH generally forms itself as a free radical component. Ascorbic acid, isoeugenol, and isoascorbic acid can make DPPH reach to a steady state by reacting with it, as they have antioxidant properties [62, 63]. FRAP is another assay method to detect the antioxidant property of an unknown compound. Here, ferrous ion forms by the reduction of ferric ion and generates a colored complex of ferrous tripyridyltriazine at low pH. By comparing the absorbance between the test reaction mixture and the mixture of known concentrations containing ferrous ion, FRAP values are obtained [64]. Another popular antioxidant assay method is CUPRAC (cupric reducing antioxidant capacity), where copper (II)-neocuproine reagent works as the chromogenic oxidant [65]. Nitric oxide (NO) scavenging capacity is another test for antioxidants that is often used for *in vitro* research. The basic mechanism of it is the reaction of the antioxidant with NO, which is characterized by the electron transfer from NO to the antioxidant [66].

We observed that the methanolic extract of A. philoxeroides seemed to have significantly higher antioxidant activity than the standard in all four antioxidant assay techniques. In our experiment, the IC_50_ values for DPPH and NO free radical scavenging capabilities of A. philoxeroides extract were 116.63 *μ*g/ml and 176.74 *μ*g/ml, respectively. Meanwhile, the IC_50_ values for DPPH and NO free radical scavenging capacity of *A. philoxeroides* extract were 443.38 0.38 *μ*g/ml and 228.11 0.61 *μ*g/ml, respectively, in a previous work done by Ramproshad, et al. Both findings suggest a positive relationship between phenolic and antioxidant activity, implying that when total phenolic levels rise, antioxidant activity rises as well [67]. However, the relationship might sometimes fluctuate due to the type of phenolics and the amount of each phenolic component present in the sample [11]. As a result, more research is needed to figure out which phenolic chemicals are responsible for the species' antioxidant activity and how they contribute to it. To establish the potential application of these species in pharmaceutical treatment, further in vivo antioxidant investigations are warranted.

Besides that, our findings disclosed *that A. philoxeroides* has a modest concentration-dependent ferric and cupric reducing ability when compared to ascorbic acid (Figures 2(c), 2(d)). We were unable to compare our outcomes with other studies since we were unable to get any research on antioxidant activity based on ferric and capric reduction potential of the examined plant species. Our observations, however, are similar to those of other members of this family, such as *Alternanthera sessilis (Linn)* [68].

Anticoagulant activity can be determined through different assay methods. Prothrombin time (PT) and activated partial thromboplastin time (aPTT) are two well-known anticoagulant property tests. PT stands for plasma clotting time in the extrinsic coagulation cascade, while aPTT stands for intrinsic factors (factors II, V, VIII, IX, XI, and XII) and coagulation cascade clotting time [69, 70]. We determined a significant anticoagulant activity of *A. philoxeroides* (Table 3). Therefore, it can be suggested as an effective anticoagulant agent against abnormal blood coagulation or a hypercoagulable state. Besides the evaluation of anticoagulant activity of the sample extract (*A. philoxeroides*), the anticoagulant activity of the HPLC identified bioactive compounds was also measured (Table 4). It was done to see the biological effect as an anticoagulant agent of bioactive compounds which were detected. The anticoagulant activity of the detected bioactive compounds (i.e., tannic acid, catechin, gallic acid, and vanillic acid) was measured through PT and aPTT tests.

Focusing on the anticoagulant activity of the bioactive compounds (Table 4), we discovered that vanillic acid had the strongest anticoagulant influence in the case of PT, followed by gallic acid, tannic acid, and catechin. However, in the case of aPTT, we found that the activity of tannic acid was the most prominent, followed by catechin, gallic acid, and vanillic acid. Nevertheless, all detected bioactive compounds showed anticoagulant activity, although the strength was not the same.

As vanillic acid showed the highest activity in PT and the lowest activity in aPTT among these four bioactive compounds, and it is possible that it will be highly effective in the extrinsic coagulation cascade and will have a poor effect on intrinsic coagulation factors or the cascade of the blood coagulation process. Gallic acid's relatively strong PT activity and considerably reduced aPTT activity indicates its potential effectiveness in the extrinsic coagulation cascade compared to the intrinsic pathway. Analyzing the PT and aPTT values, tannic acid seemed to be exceedingly effective in the intrinsic factors or pathway of coagulation as compared to the extrinsic pathway among all four bioactive compounds. Catechin demonstrated the lowest PT value which proved itself as unable to compete with three other bioactive compounds in case of showing efficacy in the extrinsic pathway of coagulation. Nevertheless, its aPTT value reveals its significant possibility and efficacy in the intrinsic coagulation cascade. These findings indicated that *A. philoxeroides* had anticoagulant capabilities and could be used to treat patients who are in need of anticoagulation therapy [71, 72].

A shift in the body's typical weight implies that distinct body organs are no longer functioning properly [73]. During the 4-week treatment period, the change in the rat's body weight was insignificant as compared to the control group (Figure 3). This extract appears to have no adverse effects on the body weight of mice which were given three different dosages. Likewise, in humans and other species, relative organ weight can be used to determine various physiological and pathological states [74]. Toxicants cause unexpected metabolic responses in the body's major organs [75]. Our findings concluded that the three doses of *A. philoxeroides* methanol extract (250 mg/kg, 500 mg/kg, and 1000 mg/kg) are nontoxic to the internal organs, as daily administration of *A. philoxeroides* extract had no discernible effect on relative organ weights (heart, liver, kidney, lung, stomach, and brain) when compared to the control group. As a result, this extract of *A. philoxeroides* is regarded to be safe for preserving major organ functions.

What is more, no visible lesions, such as inflammation, vacuolization, massive separation, or necrosis in the principal organ systems, were observed at doses up to 1000 mg/kg. Histological findings confirmed that *A. philoxeroides* extract was well adjusted with the heart, liver, kidney, lung, stomach, and brain tissue of mice at 250, 500, and 1000 mg/kg. Tannic acid, gallic acid, catechin, and vanillic acid may exert a nontoxic effect. These findings will be beneficial in directing people throughout the world to consume this high-antioxidant plant variety. However, the quality of the soil in which *A. philoxeroides* was cultivated should be considered before purchasing consumption, as it is an effective accumulator of heavy metals such as Fe, Zn, Mn, Pb, and Cd [76].

Evaluation of hematological parameters is valuable to assess the quantity of toxic effects of chemical compounds and herbal extracts on the blood composition of the experimental animal [77]. Numerous hematological assays in animals are carried out to identify several disease situations which seemingly look like unusual toxicity signs and symptoms of humans [78]. As erythrocyte parameters such as RBC, HGB, HCT/PCV, MCV, MCH, and RDW reveal significant evidence about anemic conditions, polycythemia and thalassemia, our results displayed no conspicuous hemolytic alterations in these parameters signifying no changes in the erythropoiesis, morphology, or osmotic fragility of RBC [79]. Similarly, an upsurge of leukocyte production is usually considered as a stress marker and a defensive mechanism in contradiction of numerous inflammatory circumstances such as polymyalgia rheumatica, bacterial infections, hemorrhage, and leukemia [79]. We found no noteworthy deviations in the leukocyte counts except for neutrophils which declined remarkably, and this result complies with the findings of Sireeratawong et al. [79]. According to de Andrade et al., neutrophil decrease might be correlated to the anti-inflammatory potentiality of the plant extract [80]. Platelets and platelet indices, such as MPV, PDW, and PCT, are also important indications for the detection of thromboembolic disorders and atherosclerosis, which increases with platelet activation time [81]. The nonsignificant effect on platelet counts hypothesizes that the extract has no contribution to induce thromboembolic disorders on the blood composition.

## 5. Conclusion

Taking the whole information into account, *A. philoxeroides* was identified having an elevated quantity of phenolic compounds, flavonoid, and tannins, and it showed a potential performance in free radical scavenging capacity with high antioxidant activity against in vitro oxidative stress. Besides, it showed a significant antimicrobial effect against both pathogenic and nonpathogenic bacteria. *A. philoxeroides* can be a significant anticoagulant agent against hypercoagulation and different diseases related to hypercoagulable states as it was investigated to increase both PT and aPTT in our study. Finally, the nontoxic impact assessed by body weight, as well as fundamental organ weight and hematological evaluation, shows that therapeutic dosages have a large margin of safety. More exploration should be carried out to identify and clarify the bioactive components, as well as to understand structural configurations with precise pharmacology for this remarkable tropical herb.

## Figures and Tables

**Table 1:** Total phenols, flavonoids, and tannin contents in *A. philoxeroides*.

Phytochemicals	Quantity present in *A. philoxeroides* (mg/g)
Total phenols (GAEs)	181.75 ± 2.47
Total flavonoids (CEs)	101.5 ± 3.53
Total tannins (TEs)	68.58 ± 0.80

Data are presented as means ± SD (standard deviation). GAE, gallic acid equivalent; CE, catechin equivalent; and TE, tannic acid equivalent.

**Table 2:** Phenolic acids identified in *Alternanthera philoxeroides*.

Standard	Retention time of standards (min)	Retention time of *A. philoxeroides* (min)	Concentration (mg/g)
Tannic acid	3.781	3.533	0.248
Gallic acid	4.297	3.963	0.334
Catechin	5.202	5.111	0.091
Vanillic acid	5.998	5.720	0.278
Rutin	6.599	ND	ND
Quercetin	7.533	ND	ND

Here, ND = not detected.

**Table 3:** Anticoagulant activities of *A. philoxeroides* extract (PT and aPTT).

Anticoagulant test	Control clotting time	Clotting time in the presence of *A. philoxeroides* extract at various concentrations (*μ*g/ml)		
		250 *μ*g/ml	500 *μ*g/ml	1000 *μ*g/ml
PT	9.53	10.16	10.68	13.26
aPTT	55.92	56.94	60.82	66.28

Here, the values are expressed in time (sec.).

**Table 4:** Anticoagulant activity of the bioactive chemicals derived from *A. philoxeroides*

Bioactive chemicals	Control PT	PT values of bioactive chemicals (10 *μ*g/ml)	Control aPTT	aPTT values of bioactive chemicals (10 *μ*g/ml)
Tannic acid	9.53	12.62	55.92	57.54
Gallic acid		13.33		56.26
Catechin		11.71		56.87
Vanillic acid		15.91		56.14

Here, the values are expressed in time (sec.).

**Table 5:** Influence of three dosages of *A. philoxeroides* methanol extract on the blood parameters of mice.

	Hematological parameters	Groups			
		Control	*A.philoxeroides* extract (250 mg/kg)	*A.philoxeroides* extract (500 mg/kg)	*A.philoxeroides* extract (1000 mg/kg)
Erythrocyte count	RBC (m/uL)	2.52 ± 0.90	5.70 ± 2.95	3.61 ± 0.25	3.73 ± 1.80
	HGB (g/dL)	13.15 ± 0.05	12.95 ± 0.25	13.50 ± 0.20	12.35 ± 0.55
	HCT/PCV (%)	10.45 ± 3.85	24.95 ± 13.05	15.60 ± 1.00	16.35 ± 7.95
	MCV (fL)	41.30 ± 0.60	43.65 ± 0.35	43.25 ± 0.25	43.70 ± 0.20
	MCH (pg)	59.95 ± 21.55	31.35 ± 16.65	37.55 ± 2.05	42.20 ± 18.90
	RDW (%)	19.90 ± 0.90	16.70 ± 3.80	21.65 ± 0.05	17.80 ± 0.80

Leukocyte count	WBC	7600 ± 1100	4750 ± 2350	3050 ± 1550	4750 ± 2850
	Lymphocyte (%)	67.00 ± 1.00	92.00 ± 4.00	81.00 ± 7.00	86.00 ± 5.00
	Monocyte (%)	1 ± 1	1.5 ± 0.5	0 ± 0	0 ± 0
	Neutrophil (%)	23.50 ± 1.50	6.00 ± 4.00^*∗*^	4.50 ± 2.50^*∗*^	8.00 ± 2.00^*∗*^
	Basophil (%)	0 ± 0	0 ± 0	0.5 ± 0.5	1 ± 0
	Total cir. eosinophil Count (TEC) (/cumm)	847 ± 197	24 ± 0	365 ± 95	57 ± 0

Platelet count	Total platelet count (PC) (/cumm)	2309500 ± 225500	1298000 ± 563000	1787500 ± 176500	1622000 ± 307000
	MPV (m^3)	11.10 ± 0.10	9.40 ± 0.70	10.80 ± 0.20	9.80 ± 0.70
	PDW (%)	28.75 ± 3.45	30.7 ± 7.1	24.85 ± 3.55	23.8 ± 8
	PCT (%)	2.56 ± 0.23	1.26 ± 0.62	1.93 ± 0.23	1.57 ± 0.19

The results are presented as the mean ± SD of six animals in each group. The mean values with different superscript (^*∗*^) on the same row denote significance differences compared to the control (*p* < 0.05). This dataset was analyzed using a one-way ANOVA followed by Dunnett's multiple comparisons.

## Data Availability

Data are available on request.

## Supplementary Materials

Supplementary Materials Supplementary File 1 provides an explanation of the methods used to measure the total phenol, flavonoid, and tannin levels.
