# Supplementary material for: Subchronic Toxicity Study of Alternanthera philoxeroides in Swiss Albino Mice Having Antioxidant and Anticoagulant Activities
Source: J Toxicol. 2022 Jul 13;2022:8152820. doi: 10.1155/2022/8152820 (PMC9300360; doi:10.1155/2022/8152820)
Supplement: Supplementary Materials — Supplementary File 1 provides an explanation of the methods used to measure the total phenol, flavonoid, and tannin levels. [file 8152820.f1.docx]

# Phytochemical analysis

## Total Phenol

Total phenol, flavonoid and tannin content were determined respectively. Phenol content was determined following an established method based on Folin-Ciocalteu Reagent (FCR) using 0.4 ml of extract (2 mg/ml) and different concentrations (12.5-200 µg/ml) of standard (Gallic acid) [[1](#_ENREF_1)]. Sodium carbonate solution (1.6 ml, 7.5%) followed by Folin-Ciocalteu reagent solution (5 ml, 10 fold diluted) were taken into each tube and incubated in the dark for approximately 1 hour and finally the absorbance was measured at 765nm. Total phenol contents of the fractions were expressed as Gallic acid equivalents (GAE).

## Total Flavonoid

Total flavonoid content was determined using Aluminium chloride colorimetric assay method described by Chang and Yang [[2](#_ENREF_2)] with slide modification. 1.0 ml of sample extract (0.25 mg/ml) and various concentrations (5-80 µg/ml) of Catechin as the standard were taken in different test tubes followed by the addition of distilled water (4 mL) and sodium nitrite (0.3 ml, 5%). Aluminium chloride (0.3 mL, 10%) followed by sodium hydroxide (2 mL, 1M) were added after 5 and 6 minutes respectively and distilled water (2.4 mL) was added immediately. Finally, the absorbance was measured at 510 nm. Total flavonoid content of the sample extract was expressed as Catechin equivalents (CE).

## Total tannin

The Folin-Ciocalteu method [[3](#_ENREF_3)] was used to determine the total tannin content, where 7.5 ml of distilled water was mixed with sample extract (0.1 mL, 0.5 mg/mL) and various concentrations (6.25-100 µg/ml) of the standard (tannic acid). Folin-Ciocalteu reagent (0.5 mL) was added to each tube followed by sodium carbonate (1 mL, 35%) and distilled water (0.9 mL). Finally, the solution mixture was incubated for 30 minutes and the absorbance was measured at 725 nm. Tannin content of the sample extract was expressed as tannic acid equivalent (TE).

# References

1. Singleton, V.L., R. Orthofer, and R.M. Lamuela-Raventós, *Analysis of total phenols and other oxidation substrates and antioxidants by means of folin-ciocalteu reagent*, in *Methods in enzymology*. 1999, Elsevier. p. 152-178.

2. Chang, C.-C., et al., *Estimation of total flavonoid content in propolis by two complementary colorimetric methods.* Journal of food and drug analysis, 2002. **10**(3).

3. Tambe, V.D. and R. Bhambar, *Estimation of total phenol, tannin, alkaloid and flavonoid in Hibiscus tiliaceus Linn. wood extracts.* RRJPP, 2014. **2**(4): p. 41-47.
